# Applications of emerging extracellular vesicles technologies in the treatment of inflammatory diseases

**DOI:** 10.3389/fimmu.2024.1364401

**Published:** 2024-03-13

**Authors:** Kecheng Lou, Hui Luo, Xinghua Jiang, Shangzhi Feng

**Affiliations:** ^1^ Department of Urology, Jiujiang University Clinic College/Hospital, Jiujiang, Jiangxi, China; ^2^ Department of Urology, Lanxi People’s Hospital, Jinhua, Zhejiang, China; ^3^ The First Clinical College, Gannan Medical University, Ganzhou, Jiangxi, China; ^4^ Department of Urology, Jingdezhen Second People’s Hospital, Jingdezhen, Jiangxi, China

**Keywords:** extracellular vesicles, inflammatory, plant-derived EVs, milk-derived EVs, MSC-derived EVs, macrophage-derived EVs

## Abstract

The emerging extracellular vesicles technologies is an advanced therapeutic approach showing promising potential for addressing inflammatory diseases. These techniques have been proven to have positive effects on immune modulation and anti-inflammatory responses. With these advancements, a comprehensive review and update on the role of extracellular vesicles in inflammatory diseases have become timely. This review aims to summarize the research progress of extracellular vesicle technologies such as plant-derived extracellular vesicles, milk-derived extracellular vesicles, mesenchymal stem cell-derived extracellular vesicles, macrophage-derived extracellular vesicles, etc., in the treatment of inflammatory diseases. It elucidates their potential significance in regulating inflammation, promoting tissue repair, and treating diseases. The goal is to provide insights for future research in this field, fostering the application and development of extracellular vesicle technology in the treatment of inflammatory diseases.

## 1 Introduction

Epidemiological studies indicate a significant increase in the incidence of inflammatory diseases over the past 20 years (1). Consequently, the total number of patients taking immunosuppressive drugs continues to rise (2). Prolonged use of immunosuppressive drugs inevitably increases the risk of infections and malignancies as antimicrobial and antitumor immunity remains suppressed (3). When the normal inflammatory process fails to resolve, chronic inflammatory pain ensues, leading to an excess of pro-inflammatory cytokines and chemotactic factors, ultimately resulting in central sensitization (4–6). Therefore, there is an urgent need for new therapeutic drugs in the treatment of autoimmune and inflammatory diseases, capable of suppressing detrimental immune responses without inducing life-threatening immunosuppression.

**Figure 1:**
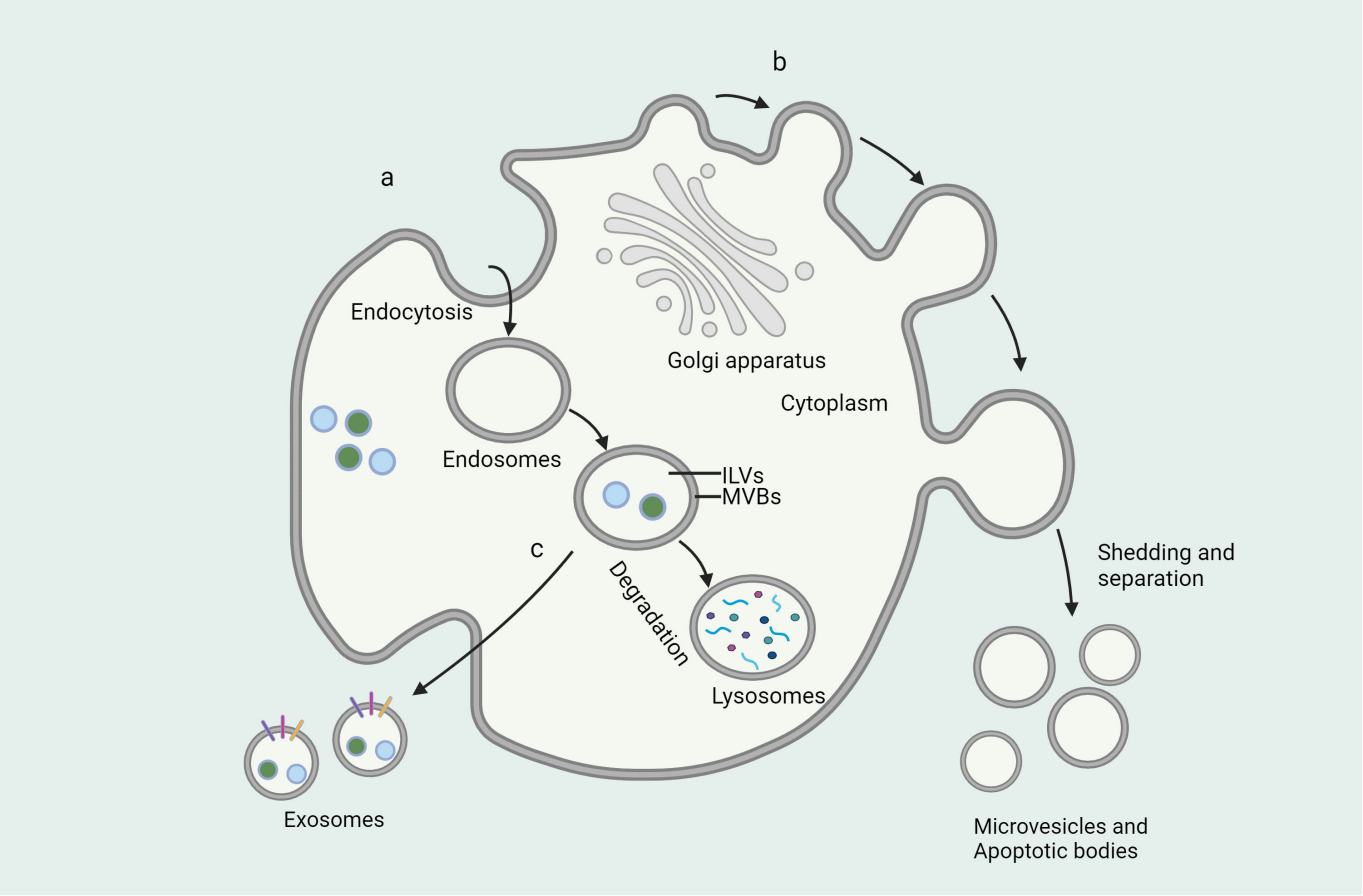
Formation of extracellular vesicles and mode of secretion. **(A)** Endosomes are vesicles formed by the cell membrane surrounding a substance and formed by endocytosis, where the substance is broken down by fusion with lysosomes or released outside the cell by direct fusion with the cell membrane. **(B)** Microvesicles are vesicles formed by the direct shedding of cell membranes through shedding and separation of cell membranes. **(C)** Exosomes are vesicles produced through the endoplasmic reticulum and Golgi apparatus that fuse with the cell membrane and release them outside the cell.

**Figure 2:**
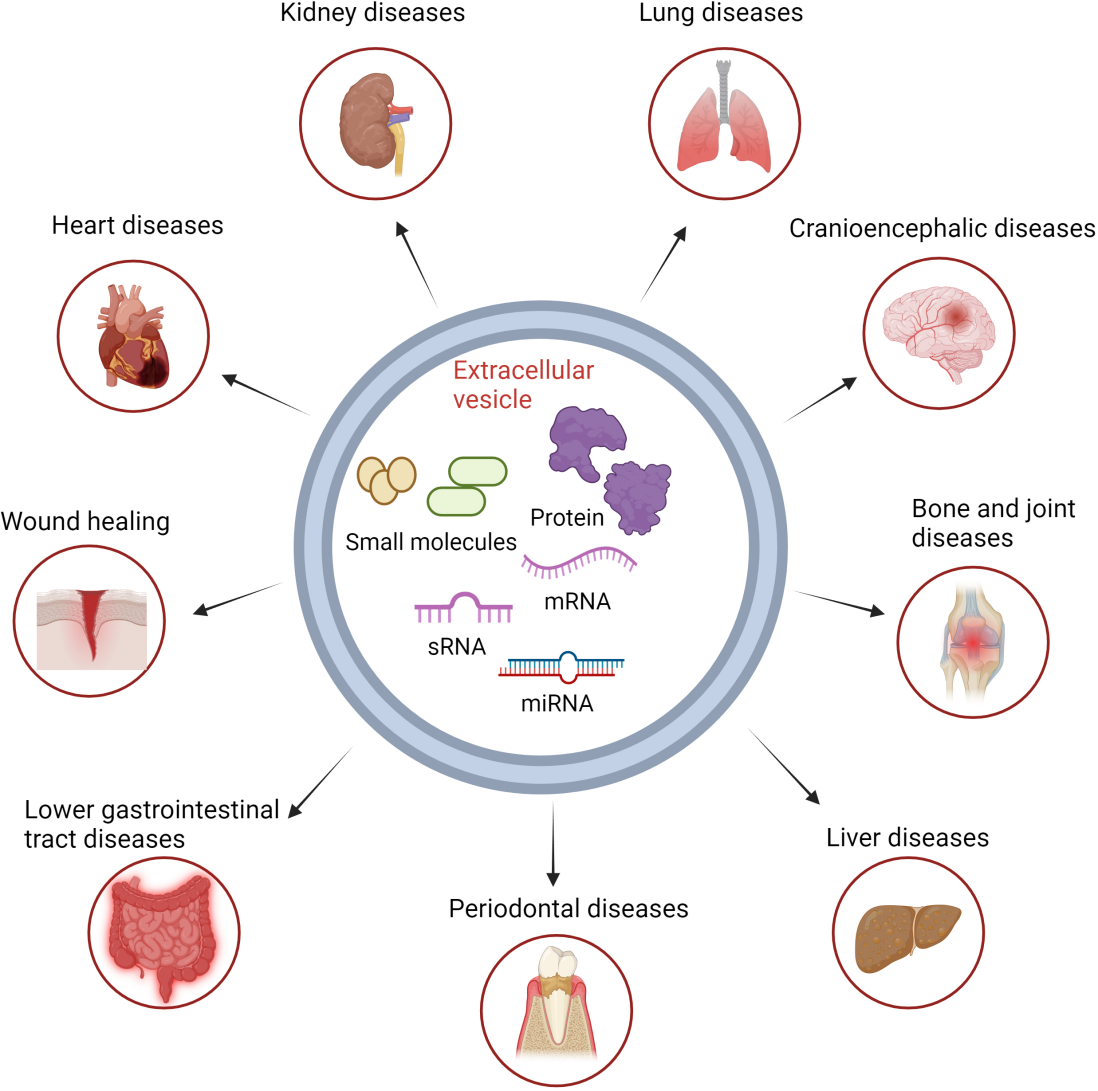
Basic structure of extracellular vesicles and the treatment of immune-related diseases. Biomolecules in extracellular vesicles can be directly transported by vesicle targeting to the target cells, resulting in therapeutic effects for inflammatory diseases.

Extracellular vesicles (EVs) are a class of small cellular products with sizes in the nanometer range and are found to possess a bilayer membrane structure. These small cellular products are released by all types of cells under both normal and pathological conditions (7)(**Figure 1**). EVs contain a rich array of biomolecules such as proteins, lipids, RNA, cellular metabolites, as well as active molecules like growth factors and cytokines (8). Simultaneously, EVs as nanocarriers offer advantages over other systems due to their ability to carry various endogenous biomolecules, exhibiting biocompatibility, natural targeting capabilities, and evasion from clearance (9, 10) (**Figure 2**). Recently, increasing attention has been focused on the therapeutic role of emerging extracellular vesicle technology in inflammatory diseases. Recent studies have highlighted the prospects of extracellular vesicle technology in the treatment of inflammatory diseases. This emerging technology has garnered widespread attention not only for disease treatment but also for its involvement in immune system regulation. By exploring the potential role of extracellular vesicles in immune modulation, we may break the constraints of conventional treatments, presenting new opportunities for the diagnosis and treatment of inflammatory diseases. These findings offer unprecedented possibilities for the exploration of future therapeutic tools and approaches.

## 2 Novel extracellular vesicles in inflammatory diseases

The role of extracellular vesicles in the study of inflammatory diseases has become a hot topic in biomedical research in recent years. They are capable of carrying and delivering proteins, lipids, RNA, and DNA and thus play a role in many physiological and pathological processes, including inflammatory response, immune regulation, and disease progression (11). Compared to conventional EVs, these emerging EVs can be engineered with specific markers to improve their targeting, allowing them to more effectively reach and act on inflamed or damaged tissues (7). Based on their unique origin and biological activity, these emerging EVs offer new possibilities for personalized and tailored therapies, especially in the treatment of inflammatory diseases (12, 13). The development of these emerging extracellular vesicle technologies not only extends our understanding of the role of EVs in physiological and pathological processes, but also provides new therapeutic tools and strategies, especially in the treatment of inflammatory diseases (14). With further research, these technologies are expected to overcome the limitations in the application of traditional EVs and realize more effective and safer therapies. For example, plant extracellular vesicles, breast milk extracellular vesicles, mesenchymal stem cell extracellular vesicles, macrophage extracellular vesicles, and other novel extracellular vesicle technologies. The respective advantages of these EVs and their potential value in inflammatory diseases may help to revolutionize and solve the current dilemma of treating immune diseases. Therefore, it is necessary to discuss in detail the research and challenges of these vesicles in inflammatory diseases with a view to promoting the clinical utilization of novel extracellular vesicles.

## 3 Macrophage-derived extracellular vesicles

### 3.1 Overview

Macrophage-derived extracellular vesicles (Mφ-EVs) play a unique role in immune responses, holding potential therapeutic applications for various diseases (15). These vesicles alter the phenotype and function of target cells by carrying a rich cargo of proteins, lipids, and genetic information, although their composition may vary with different phenotypes of macrophages or microenvironments (16). Mφ-EVs modulate communication between local and systemic cells, temporally and spatially regulating molecular events in recipient cells, such as promoting the resolution of inflammation and alleviating inflammation-induced allergic reactions (17). The surface of Mφ-EVs is enriched with immune molecules like cluster of differentiation 47 (CD47), enabling them to evade immune surveillance and evade immune attacks (18). Compared to larger macrophages, extracellular vesicles are smaller, more easily circulated, and possess the capability to traverse biological barriers, thus can be further engineered for potential drug delivery systems (19).

### 3.2 Applications as therapeutic agents

The RNA molecules (20, 21), protein components (22, 23), soluble mediators (such as enzymes and cytokines) (24, 25), and lipids (26) found in EVs secreted by macrophages can stimulate pro-inflammatory signal transduction and activate receptor immune cells, resulting in the formation of a local immune-stimulating microenvironment (17, 27, 28). They exhibit a strong inclination and high affinity for tumors and inflammatory tissues (29, 30). Mφ-EVs have three main phenotypes: unpolarized M0-derived EVs (M0-EVs), M1-derived EVs (M1-EVs), and M2-derived EVs (M2-EVs). Their biological functions differ based on the characteristics of the parent cells (31). M1-type macrophage-derived extracellular vesicles (M1-EVs) play a crucial role in heart repair and remodeling by transporting miR-222 and miR-155, regulating stem cell apoptosis, inflammatory responses, and vascular regeneration abilities (32–36). In contrast, M2-type macrophage-derived extracellular vesicles (M2-EVs) execute cardiac repair functions by transferring miR-1271-5p, inhibiting cardiomyocyte apoptosis (37). In ischemia-reperfusion injury (IRI), M1-type macrophage-derived extracellular vesicles (M1-EVs) promote cardiomyocyte necrosis through miR-29a, resulting in cardiac dysfunction, while M2-type macrophage-derived extracellular vesicles (M2-EVs) alleviate IRI by miR-148a, suppressing the activity of specific signaling pathways to mitigate cardiac injury (38, 39). M1-type macrophage-derived extracellular vesicles (M1-EVs) play a critical role in chronic low-grade tissue inflammation-induced insulin resistance, where their cargo miRNAs (such as miR-212-5p, miR-155, and miR-29a) target specific genes or modulate signaling pathways, limiting insulin secretion, impairing insulin’s suppression of glucose, potentially contributing to pancreatic β-cell failure, and obesity-induced insulin resistance (40–42). Both M1 and M2-type macrophage-derived extracellular vesicles have distinct roles in diabetic complications. M1-EVs aid in accelerating wound healing, modulating Mφ phenotypes, inhibiting osteogenesis, while potentially inducing inflammatory reactions (43–45). On the other hand, M2-EVs alleviate foot lesions, promote mesangial cell proliferation in glomeruli, yet are associated with inflammation and mitochondrial dysfunction (46–48). This underscores the intricate, sometimes contradictory, roles of EVs in the pathophysiology of diabetes. However, due to the complex and mixed phenotypes typically exhibited by macrophages in different diseases or stages within a disease, even within the same disease, identifying their exact subgroups of EVs (such as M1 or M2) is challenging.

### 3.3 Applications as therapeutic carrier

Due to the inherent ability of macrophage-derived EVs to traverse natural barriers within the body, they can selectively deliver payloads to hard-to-reach sites while minimizing side effects on healthy tissues (49, 50). Zhang et al. isolated EVs from umbilical cord blood of M1Mφ and M2Mφ loaded with cisplatin (CDDP). Results showed that compared to M2-EVs, M1-EVs exhibited higher cytotoxicity in drug-resistant cisplatin-resistant ovarian cancer cell line (A2780/DDP) cells, suggesting their potential as drug delivery tools in drug-resistant environments (51). Moreover, Mφ-EVs transport neuroprotective factors across the blood-brain barrier, demonstrating neuroprotective effects (49). Novel Mφ-EV delivery systems exhibit inhibitory effects on oxidative stress and inflammation, potentially aiding in the treatment of neurological disorders (52). Furthermore, Mφ-EVs carrying specific drugs show potential in enhancing drug targeting and promoting drug bioavailability in the brain, holding promise in reducing neuronal damage and promoting neuronal protection (53, 54). Gao et al. discovered that M2-EVs delivering berberine drugs enhanced drug targeting, prolonged duration, and impacted inflammation and cell apoptosis, prompting a shift of Mφ from an inflammatory to a healing phenotype (55). Similarly, Mφ-EVs loaded with baicalin improved drug solubility and brain targeting, providing significant neuroprotection for ischemic stroke patients (56). These findings offer new therapeutic strategies utilizing Mφ-EVs as drug delivery systems, opening new avenues for the treatment of neurological disorders.

## 4 Plant-derived extracellular vesicles

### 4.1 Overview

Plant-derived extracellular vesicles are nanosized vesicles secreted by plant cells, sharing similar characteristics to extracellular vesicles from animal cells. They originate from various edible plants and play roles in various applications through different mechanisms (57). One significant application is in the treatment of inflammatory diseases, where they can enhance the immune system’s resistance to diseases and serve as nanocarriers for therapeutics (58). Engineered plant-derived extracellular vesicles exhibit excellent targeting, nanoscale dimensions, low off-target effects, low toxicity, and have the potential for large-scale production, offering advantages in the field of drug delivery (59–61). However, due to significant variations in the quantity, size distribution, antioxidant activity, and other characteristics of plant-derived extracellular vesicles among different plant species, and influenced by the chemical properties of the source plants, separate evaluations are needed for the safety and effectiveness of each type of extracellular vesicle (62).

### 4.2 Applications as therapeutic agents

When discussing treatment strategies for inflammatory diseases, plant-derived extracellular vesicles have garnered significant attention as an effective therapeutic agent. They possess the ability to exert anti-inflammatory effects through various mechanisms such as modulating the immune system, inhibiting inflammatory factors, and clearing oxidative stress. Their role in the treatment of colitis, in particular, has been confirmed by numerous studies (63–67).

As an example, plant-derived extracellular vesicles have demonstrated significant therapeutic effects in numerous experiments concerning colitis. Nanoparticles derived from broccoli can inhibit colitis in mice by activating dendritic cell adenosine monophosphate-activated protein kinase (AMPK) (68). Grape-derived extracellular vesicles promote proliferation and differentiation of intestinal stem cells, facilitating intestinal epithelial regeneration and protecting mice from dextran sulfate sodium (DSS)-induced colitis (57). Ginseng-derived extracellular vesicles suppress the nuclear factor Kappa-Light-Chain-Enhancer of activated B cells (NF-κB) pathway, reduce inflammatory factor levels, and improve gut microbiota composition, effectively treating DSS-induced colitis in mouse models (69, 70). Lemon-derived extracellular vesicles manipulate probiotics (C. diff) to inhibit Clostridioides difficile infection, a major cause of antibiotic-associated colitis (71, 72). Moreover, ginger and ginger rhizome-derived extracellular vesicles not only inhibit colitis but also exhibit inhibitory effects on nucleotide-binding oligomerization domain (NOD)-like receptor protein 1 (NLRP1) inflammasome activation (59, 73, 74). Mulberry bark-derived extracellular vesicles also protect mice from colitis by activating the Aryl hydrocarbon receptor (AhR) signaling pathway mediated by heat shock protein family A member 8 (HSPA8) (75).

Furthermore, the potential applications of plant-derived extracellular vesicles in treating other inflammatory diseases are increasingly evident. For instance, extracellular vesicles from ginger exhibit significant efficacy in preventing and treating chronic periodontitis, primarily attributed to specific interactions between phosphatidic acid and miR-159a-3p in the vesicles and Hemin-binding protein 35 in P. gingivalis (76). Ginger-derived extracellular vesicles also target Severe Acute Respiratory Syndrome Coronavirus 2 nonstructural protein 12 (SARS-CoV-2 Nsp12) and spike genes to ameliorate lung inflammation and act through the Toll-like receptor 4 (TLR4)/TIR-domain-containing adapter-inducing interferon-β (TRIF)-dependent pathway in NF-E2-related factor 2 (Nrf2) induction, preventing alcohol-induced liver injury (77, 78). Extracellular vesicles from other sources like honey and shiitake mushrooms also inhibit the activation of the nucleotide-binding domain, leucine-rich-containing family, pyrin domain-containing 3 (NLRP3) inflammasome, reducing inflammation and liver damage in experimentally induced acute liver injury (79, 80). Currently, three plant-derived extracellular vesicles from grapes, ginger, and aloe vera are undergoing clinical trials related to inflammatory diseases (81). With more clinical trials underway, we may gain further insights into the role of plant-derived extracellular vesicles in the human body, aiding in a more accurate assessment of their potential and value in clinical applications.

### 4.3 Applications as therapeutic carrier

In recent years, plant-derived extracellular vesicles have been discovered to possess the capability of carrying drugs to target tissues, holding immense potential in treating inflammatory diseases. These vesicles are considered ideal nano-carriers as they contain common proteins, bioactive lipids, RNA, and other pharmacologically active molecules involved in transfer and transport (58, 82). Sung et al.’s research demonstrates that extracellular vesicles derived from ginger, when orally administered, effectively target the colon, reducing the incidence of acute colitis, enhancing intestinal repair capabilities, and preventing the onset of chronic colitis and colitis-associated cancers (83). Grapefruit-derived extracellular vesicles also exhibit similar abilities, efficiently delivering therapeutic agents to inflammatory tumor sites by activating leukocyte migration pathways (84, 85). More excitingly, when combining the anti-inflammatory drug methotrexate (MTX) with glucocorticoid-induced tumor necrosis factor receptor family-related protein (GNV), it significantly reduces the toxicity of MTX and enhances its therapeutic effects in inflammatory diseases (86). The advantages mentioned above endow plant extracellular vesicles with enormous potential as a novel drug delivery system. Researchers successfully utilized lipids extracted from extracellular vesicles sourced from ginger to prepare ‘natural’ nano-carriers. These carriers loaded with siRNA for treating ulcerative colitis demonstrated superior effects compared to traditional synthetic nanoparticles (74, 87, 88). Furthermore, a crucial characteristic of plant extracellular vesicles as drug delivery carriers is their capacity for personalized adjustments based on treatment targets. For instance, grape-derived extracellular vesicles can target intestinal cells, while grapefruit-derived extracellular vesicles can target macrophages (57, 86). This offers possibilities for personalized therapeutic approaches.

## 5 Mammary gland-derived extracellular vesicles

### 5.1 Overview

Mammary gland-derived extracellular vesicles (mEVs) are tiny vesicles originating from mammary epithelial cells, released into milk through endosomal pathways or directly from the cell membrane (89, 90). These mEVs contain a plethora of immune-related microRNAs and proteins, observed not to induce systemic toxicity or adverse immune reactions (91, 92). Breast milk is a rich source of miRNAs, where miRNAs play a crucial role in post-transcriptional gene regulation and may impact cellular gene expression through the presence of lactation-specific and immune-related proteins and miRNAs (93–95). These beneficial mEVs have been found not only in human milk but also in cow and goat milk (96). They carry beneficial miRNAs capable of entering cells and modulating biological functions such as promoting cell proliferation, regulating inflammatory responses, protecting cells from damage, and aiding in tissue functional recovery (97–99).

### 5.2 Applications as therapeutic agents

Mammary gland-derived extracellular vesicles carry unique RNA, proteins, lipids, and DNA, possessing anti-degradation, antioxidant, and anti-inflammatory biological properties (100). Studies have found these vesicles to play a crucial role in the treatment of intestinal inflammation and colitis. They can enhance the vitality and proliferation capacity of intestinal epithelial cells (97), improve intestinal barrier function (99), and inhibit inflammatory signal transduction (101). Additionally, mammary gland-derived extracellular vesicles can reduce intestinal epithelial damage, restore intestinal tight junction proteins, and regulate inflammation and cellular homeostasis, which holds significance for inflammatory bowel diseases such as necrotizing enterocolitis and inflammatory bowel disease (102, 103). The latest research indicates that mammary gland-derived extracellular vesicles can modulate lipid and amino acid metabolism in healthy mice and may alter the metabolomic characteristics of DSS-induced colitis in mice, particularly increasing levels of lipid anti-inflammatory metabolites and decreasing levels of fecal amino acids, which could be a primary driving force in alleviating colitis (100). Moreover, these vesicles have demonstrated potential applications in other inflammatory disease domains. Studies suggest their potential use in treating inflammatory lung diseases induced by agricultural dust exposure and alleviating cartilage degradation metabolism and inflammation processes in osteoarthritis (104, 105).

### 5.3 Applications as therapeutic carrier

Mammary gland-derived extracellular vesicles are considered potential drug delivery carriers due to their low immunogenicity, good biocompatibility, stability, and the ability to traverse the gastrointestinal barrier (106). Studies have found them particularly effective in the treatment of colitis (100). Furthermore, mammary gland-derived extracellular vesicles have shown potential as carriers for siRNA drug delivery. They can withstand harsh environments during digestion, improve intestinal permeability, and protect the payload (107). Because they can survive under the strongly acidic conditions of the stomach and the degrading conditions of the intestine and traverse biological barriers to reach targeted tissues, they are considered promising natural drug carrier tools for oral administration (108). These vesicles can also serve as delivery systems to enhance the bioavailability and efficacy of miRNA therapy. For instance, they can act as drug carriers for potential therapeutic miR-31-5p in diabetic wound healing (109). They can limit the degradation metabolism and inflammatory processes in cartilage by transferring growth factors and genetic modulators like miR-148a, thereby reducing cartilage damage in osteoarthritis patients (110). Another study assessed the use of mammary gland-derived extracellular vesicles as carriers for extracellular RNA therapeutics, finding breast milk to be a cost-effective source of extracellular vesicles suitable as nanocarriers for functional miRNA, potentially applicable in RNA-based therapies (111). However, the complex content and quality control of mammary gland-derived extracellular vesicles hinder their application in drug delivery (106). Further research should focus on developing novel purification and isolation techniques to address quality control issues, reduce batch-to-batch heterogeneity, and promote the application of mammary gland-derived extracellular vesicles in the field of drug delivery.

## 6 Mesenchymal stem cell-derived extracellular vesicles

### 6.1 Overview

Mesenchymal stem cells (MSCs), derived from various tissues, possess pluripotency, can differentiate into various tissues, and have regenerative capabilities (112). They not only treat tissue injuries but also exhibit immunomodulatory functions by migrating to inflammatory sites and utilizing extracellular vesicles to regulate immune responses (113). These EVs are small vesicles secreted by cells, facilitating intercellular communication through membrane transfer and various substances (114). Compared to MSCs, mesenchymal stem cell-derived extracellular vesicles (MSC-EVs) are easier to obtain and store. Furthermore, MSC-EVs are believed to pose no safety issues in cell-based therapies, such as the tumorigenic potential associated with cell administration (115, 116). Increasing evidence suggests that MSC-EVs play a significant role in immunoregulation. They contain various anti-inflammatory substances and regulate immune responses by interacting with immune effector cells (117).

### 6.2 Applications as therapeutic agents

MSC-EVs have been confirmed in numerous preclinical studies to have a positive impact on the treatment of liver diseases. Studies have shown that MSC-EVs improve liver inflammation and alleviate various liver diseases by modulating immune responses. MSC-EVs have demonstrated positive effects in the treatment of non-alcoholic fatty liver disease (NAFLD) (118–122), autoimmune hepatitis (123–125), acute liver failure (126–130), liver fibrosis (131–133), and liver ischemia-reperfusion injury (IRI) (134–136). They can regulate macrophage activation, alter cytokine expression, and influence related signaling pathways.

MSC-EVs in myocardial infarction reperfusion therapy can increase ATP levels, reduce oxidative stress, and activate the Phosphatidylinositol 3-kinase/protein kinase B (PI3K/Akt) pathway to enhance myocardial vitality and prevent adverse remodeling after myocardial ischemia/reperfusion injury (137). In the Ischaemia/Reperfusion (I/R) model of myocardial ischemia/reperfusion, injection of MSC-EVs significantly reduces cell apoptosis and myocardial infarct size while improving cardiac function (138). In the field of neuroscience, researchers found that extracellular vesicles from MSCs can restore synaptic dysfunction and regulate inflammatory responses by modulating miR-21, thus enhancing learning and memory abilities in amyloid precursor protein/presenilin 1 transgenic (APP/PS1) mice (139). In the treatment of colitis, extracellular vesicles secreted by bone marrow mesenchymal stem cells (BMSCs) exhibit a positive therapeutic effect in colitis. They promote macrophage proliferation and suppress inflammation, showing positive effects in improving symptoms of ulcerative colitis and reversing experimental colitis (140, 141). More specific studies have also revealed that MSC-EVs transfer various nucleic acids, proteins, and lipids from parent cells to recipient cells, participating in chronic inflammation and immune processes, playing a significant regulatory role in the pathogenesis of arthritis (142).

### 6.3 Applications as therapeutic carrier

Research on MSC-EVs as therapeutic carriers is continually making new strides. In the treatment of idiopathic pulmonary fibrosis (IPF), bone marrow mesenchymal stem cell-derived extracellular vesicles (BMSC-EVs) and their carried microRNAs (miRNAs) show promise. Particularly, miR-186 delivered by BMSC-EVs has been found to halt the progression of lung fibrosis by inhibiting fibroblast activation, downregulating SRY-box transcription factor 4 (SOX4) and Dickkopf-related protein 1 (DKK1), offering a novel therapeutic strategy for idiopathic pulmonary fibrosis (IPF) treatment (143). In the treatment of renal diseases, mesenchymal stem cells overexpressing miRNA-let7c electively localize to damaged kidneys and upregulate the expression of miR-let7c through their secreted extracellular vesicles, thereby alleviating renal injury and significantly downregulating various fibrotic genes (144). This provides a new direction for miRNA therapy targeting renal diseases. In skin wound healing, wingless-type MMTV integration site family member 4 (Wnt4) found in human umbilical cord mesenchymal stem cell-derived extracellular vesicles is discovered to promote nuclear translocation and activity of β-catenin, enhancing proliferation and migration of skin cells (145).

In addition, MSC-EVs have played a significant role in the treatment of liver and heart diseases. MSC-EVs can carry various RNAs, exert anti-fibrotic effects, reduce the deposition of extracellular matrix (ECM), and improve liver function (146–148). In the treatment of autoimmune hepatitis, MSC-EVs carrying dexamethasone can enhance the anti-inflammatory therapeutic effect of dexamethasone while reducing its side effects (149). In heart diseases, using extracellular vesicles derived from bone marrow mesenchymal stem cells (BM-MSCs) as carriers, miR-19a/19b has been proven to significantly inhibit apoptosis of cardiac HL-1 cells (150). In myocardial infarction (MI) models, Exo/miR-19a/19b combined with MSC transplantation can significantly enhance cardiac function recovery and reduce cardiac fibrosis (150). With technological advancements, the understanding and application of MSC-derived extracellular vesicles as therapeutic carriers will continue to deepen. It can be anticipated that they will play even more critical roles in future therapeutic research.

## 7 Expectations and challenges

Emerging EVs have received widespread attention for their potential application in the treatment of inflammatory diseases as an important tool for cellular communication. EVs from different sources have demonstrated their unique properties and potential therapeutic advantages, but have also revealed common challenges that need to be overcome before clinical applications can be promoted (**Table 1**). Macrophage EVs have significant immunomodulatory capabilities and are able to ameliorate the inflammatory state by directly acting at the site of inflammation (151). However, their effects may be affected by macrophage activation status, which requires detailed phenotypic and functional analyses prior to clinical application to ensure the desired therapeutic effect. Studies of plant EVs have provided new perspectives on EVs of non-mammalian origin, which demonstrate good biocompatibility and low toxicity, making them potentially safe therapeutic vectors. However, the composition and activity of plant EVs may vary depending on the plant species and growth conditions, requiring the establishment of standardized production and purification processes to ensure product quality (152). Lactic EVs have shown unique advantages in promoting gut health and immunomodulation, especially promising applications in neonatal development. However, access to milk EVs is limited by the availability of milk sources and individual differences in their composition, factors that may affect their consistency and widespread use as therapeutic tools (153). The ability of MSC EVs to promote tissue repair and anti-inflammation makes them a powerful tool in regenerative medicine. However, challenges remain for the large-scale production and clinical application of MSC EVs, including the diversity of cell sources, standardization of the production process, and evaluation of long-term safety.

**Table 1:** Emerging extracellular vesicle technology in inflammatory diseases.

Type	Source	Function	Challenge	Reference
Macrophage-derived Evs	Macrophage	Modulates the immune response, targeting and altering the function of immune cells.	Inconclusive efficacy and potential risk of stimulating an immune storm in the organism.	(16–19)
Plant-derived Evs	Various edible plants	Highly biocompatible for use as cell-targeting nanomaterials and inexpensive for mass production of nanoparticles.	Poor single effect likelihood, potential biosafety, mixing of multiple vesicles demanding high extraction techniques.	(57–62)
Mammary gland-derived Evs	Mammary epithelial cell	Enriched with immune-modulating microRNAs/proteins to promote gut health.	The function and composition of the vesicles are influenced by the cellular state of the source animal, with the potential risk of triggering an immune storm, and their immunogenicity needs to be further investigated.	(89–91, 96, 99)
MSC-derived EVs	Mesenchymal stem cells (MSC)	Strong regeneration and repair ability, high efficiency anti-inflammatory, reduce the function of immune response.	It is expensive and not suitable for mass production, triggering the possibility of tumorization of the organism.	(112, 113, 115–117)

Each of these EVs has its own advantages in treating inflammatory diseases, but they also face different challenges. Future research needs to address how to improve the targeting of EVs, enhance their therapeutic efficacy, optimize production and purification processes, and ensure safety and stability. In addition, an in-depth understanding of the distribution, metabolism and mechanism of action of EVs *in vivo* is essential for developing customized EVs-based therapeutics and enhancing their clinical application value. On this basis, interdisciplinary collaboration has become the key to promote the research and application of EVs, pooling the strengths of biology, materials science, engineering technology, and clinical medicine to explore and develop innovative EVs-based therapeutic strategies.

## 8 Discussion

This article reviews the research findings of various types of extracellular vesicles in the treatment of inflammatory diseases. Studies have indicated that these extracellular vesicles can exert therapeutic effects by modulating immune responses, inhibiting the release of inflammatory mediators, and promoting tissue repair (14, 17, 92, 151, 152). These results affirm the potential value of extracellular vesicle technology in the treatment of inflammatory diseases.

Despite the potential therapeutic efficacy demonstrated by emerging extracellular vesicle technology in treating inflammatory diseases, it faces several challenges in practical application. Research indicates therapeutic effects of different types of extracellular vesicles in inflammatory disease models, yet obstacles persist in clinical translation. Moreover, there remain controversies and uncertainties regarding the therapeutic mechanisms, optimal dosage, effective routes, and uncertainties surrounding extracellular vesicle treatment. Deeper understanding of the generation, release, targeting mechanisms of extracellular vesicles, and enhancing their efficacy as therapeutic tools are necessary to address challenges in large-scale production, stability, and targeting. Additionally, as a drug delivery system, extracellular vesicles still require solutions for effective drug loading, improved stability and specificity, assessment of safety and effectiveness, and differences in therapeutic effects from various sources.

Future research could focus on further exploring the characteristics and mechanisms of different types of extracellular vesicles to better understand their role in the treatment of inflammatory diseases. Simultaneously, there is a need to develop more efficient production methods and improve purification techniques to enhance the yield and quality of extracellular vesicles. Additionally, finding more suitable carriers and delivery routes and conducting further clinical trials to validate their safety and effectiveness are crucial directions for future research. More interestingly, the preparation of artificial extracellular vesicles with specific targeting effects, as in the case of liposomes, may be more conducive to the large-scale clinical use of nanovesicles. Of course, this would require a more complete study of the targeting and functional mechanisms of cell-derived vesicles.

## Author contributions

KL: Writing – original draft, Writing – review & editing. HL: Investigation, Writing – original draft. XJ: Conceptualization, Writing – original draft. SF: Writing – original draft, Writing – review & editing.
